# Impact of Opioid Consumption in Patients With Functional Gastrointestinal Disorders

**DOI:** 10.3389/fphar.2020.596467

**Published:** 2020-12-21

**Authors:** Chloé Melchior, Charlotte Desprez, Fabien Wuestenberghs, Anne-Marie Leroi, Antoine Lemaire, Guillaume Goucerol

**Affiliations:** ^1^ INSERM UMR 1073, Institute for Research and Innovation in Biomedicine, Normandy University, Rouen, France; ^2^ Gastroenterology Department, Rouen University Hospital, Rouen, France; ^3^ INSERM CIC-CRB 1404, Rouen University Hospital, Rouen, France; ^4^ Physiology Department Rouen University Hospital, Rouen, France; ^5^ Gastroenterology Department, CHU UCL Namur, Godinne University Hospital, UCLouvain, Yvoir, Belgium; ^6^ Pain and Palliative Care Department, Valenciennes Hospital, Valenciennes, France

**Keywords:** opioid, tramadol, constipation, vomiting, quality of life, functional gastrointestinal disorders

## Abstract

**Objective: **We aimed to determine the burden of opioid consumption in a cohort of patients with functional gastrointestinal disorders.

**Methods: **All patients diagnosed with functional gastrointestinal disorders and referred to our university hospital were evaluated from 2013 to the beginning of 2019. Irritable bowel syndrome and functional dyspepsia diagnoses were determined according to Rome criteria and severity according to irritable bowel syndrome severity scoring system. Vomiting was quantified using a 5-point Likert scale, and constipation severity was measured using the Knowles-Eccersley-Scott-Symptom questionnaires. Quality of life was quantified by the GastroIntestinal Quality of Life Index. Patients were categorized as being treated on a chronic basis with either tramadol, step II opioids, step III opioids or as being opioid-free.

**Results: **2933 consecutive patients were included. In our cohort, 12.5% had only irritable bowel syndrome, 39.3% had only functional dyspepsia, 24.9% had a combination of both, and 23.4% had other functional gastrointestinal disorders. Among them, the consumption of tramadol, step II (tramadol excluded) and step III opioids was 1.8, 1.3 and 0.3 % respectively in 2013 and 4.3, 3.4 and 1.9% in 2018 (*p* < 0.03). Opioid consumption was associated with increased vomiting (*p* = 0.0168), constipation (*p* < 0.0001), symptom severity (*p* < 0.001), more altered quality of life (*p* < 0.0001) and higher depression score (*p* = 0.0045).

**Conclusion: **In functional gastrointestinal disorders, opioid consumption has increased in the last years and is associated with more GI symptoms (vomiting, constipation and GI severity), higher depression and more altered quality of life.

## Introduction

Opioids are mostly prescribed for acute or chronic pain associated or not with cancer referring to the World Health Organization guidelines for pain evaluation. Step III (morphine, oxycodone, fentanyl, etc.) and step II (tramadol, codeine, etc) opioid consumption increased by 45% and 65% respectively between 2006 and 2017 in the United States (Vadivelu et al., 2018). Increasing opioid prescription in the US led to 30,000 deaths due to opioid overdoses in 2015 alone (Vadivelu et al., 2018). In France, the same trend is observed with an increase of 16% from 2004 to 2017 (Chenaf et al., 2018), with a consumption associated with increased morbidity and mortality (Chenaf et al., 2018).

Opioids are known to have gastrointestinal side effects such as nausea, abdominal pain, gas and constipation (Cook et al., 2008). Among side effects, opioid-induced constipation (OIC) is the most frequent side effect and is defined by Rome IV criteria (Mearin et al., 2016). Considering their gastrointestinal (GI) side effects, and the lack of efficacy to relieve pain in functional GI disorders (FGID), opioids are not recommended in the treatment of painful FGID (Szigethy et al., 2018). On the other hand, opioids may be prescribed to treat other associated conditions in FGID patients, which may worsen their GI symptoms or trigger additional GI symptoms. However, there are currently few studies reporting opioid consumption trends in FGID patients, and the impact of opioids on GI symptoms and quality of life in FGID patients still needs to be assessed.

Therefore, our aim was to determine the burden of opioid consumption in a FGID cohort and to assess its impact on GI symptoms and quality of life.

## Material and Methods

### Study Design and Ethics

We conducted a retrospective single center study in a university hospital in Normandy, France. The study was conducted in accordance with the ethical guidelines of the Declaration of Helsinki (6th revision, 2008) and was approved by the local human research committee (E2020-51) as required by national legislation. The use of informatic data was declared to the Commission Nationale de I’Informatique et des Libertés (CNIL) (*n* 817.917), in compliance with French legislation. Written informed consent was obtained for all patients regarding the use and informatic storage of their medical data for research purposes, including score and Quality of Life questionnaires.

### Patients

All patients diagnosed with FGID and referred for GI motility tests were retrospectively evaluated from January 1st 2013 to March 18th 2019. Clinical data (age, gender, weight, height and body mass index (BMI)) were collected. Opioid consumption was evaluated by reviewing medical charts. Patients were categorized as being treated on a chronic basis with either tramadol (tramadol or an association of tramadol and paracetamol), another step II opioid (codeine, or an association of codeine and paracetamol, caffeine, or an association of caffeine and paracetamol, or an association of – codeine, caffeine and paracetamol, or an association of codeine, acetylsalicylic acid and caffeine, or an association of codeine, acetylsalicylic acid, paracetamol–, nalbuphine and opium, or an association of buprenorphine, paracetamol, opium and caffeine), a step III opioid (morphine, oxycodone, fentanyl, tapentadol) or as being opioid-free. Rheumatologic, neurologic comorbidities and migraine were assessed.

### Questionnaires

Validated self-questionnaires were systematically proposed to all patients. Irritable bowel syndrome (IBS) and functional dyspepsia (FD) were determined according to Rome criteria. Rome III criteria were used between 2013 and 2016 and Rome IV criteria since 2016 (Longstreth et al., 2006; Mearin et al., 2016). To define IBS patients before 2016, we used the association of Rome III criteria with the presence of abdominal pain (to be closest to the Rome IV definition). Severity was assessed in all patients using IBS Severity Scoring System (IBS-SSS) with a maximum score of 500 (Francis et al., 1997). Remission, mild, moderate and severe cases were defined by scores <75, 75–175, 175–300 and >300 (Francis et al., 1997).

GI symptoms were analyzed using a five-point Likert scale for vomiting (0: absent to 4: severe) and using the Knowles-Eccersley-Scott-Symptom questionnaires (KESS) for constipation (Knowles et al., 2000). KESS score comprises 11 individual items with a maximum of 39 points, a score higher than 10 defining constipation (Knowles et al., 2000).

Quality of life was quantified by the GI Quality of Life Index (GIQLI) comprising 36 questions concerning among others digestive symptoms and effects of medical treatment (Slim et al., 1999). The score range is between 0 (worst) and 144 (best quality of life) (Slim et al., 1999). Anxiety and depression were evaluated using the Hospital Anxiety and Depression (HAD) scale (Zigmond and Snaith, 1983). The scale ranges from 0: absent to 21: maximum of anxiety or depression. A score higher than 10 out of 21 defines anxiety or depression (Zigmond and Snaith, 1983).

### Statistical Analysis

Data are expressed as n (percentage) and median [Q1-Q3]. Opioid consumption was analyzed between 2013 and 2018; in 2019, patients were only included at the beginning of the year and were therefore not representative of a one-year period. Characteristics were compared using chi-squared test for qualitative variables. Non-parametric continuous variables were compared using Kruskal-Wallis test and Dunn’s test for subgroup analysis. Prevalence trends were analyzed using a chi-squared test. Associations were considered statistically significant when *p*-value was <0.05.

## Results

### Patients

Between January 1st^,^ 2013 and March 18th^,^ 2019, 3204 patients were referred for FGID. While 271 declined to give their informed consent, 2933 consecutive patients were evaluated. In our cohort, 1096 patients responded to the Rome questionnaire, 12.5% had only IBS, 39.3% had only FD, 24.9% had a combination of IBS and FD, and 23.4% had other FGID. Patients were mostly female (72.9% of the cases) with a mean age of 50.0 [36.0; 62.0] years and a mean BMI of 24.1 [21.0; 27.9] kg/m^2^. Rheumatologic, neurologic comorbidities and migraine were present in 10.4%, 6.1% and 1.5% of the patients, respectively. Patients had moderate IBS-SSS 275.0 [195.0; 339.8] with altered quality of life (GIQLI:83.0 [67.0; 99.0]). Constipation was a ubiquitous problem with a KESS score of 11.0 [7.0; 17.0] points. Patients had a mean score of HAD-anxiety of 9 [7.0; 12.0] points and of HAD-depression of 6.0 [3.0; 9.0] points. Anxiety was therefore present in 39.1% of the patients and depression in 14.9%.

Patients were on laxatives, Peripherally-Acting Mu-Opioid Receptor Antagonists (PAMORA), antiemetics, loperamide, PPI, steroids and NSAIDs in 9.8%, 0.3%, 3.7%, 5.1%, 19.7%, 1.4% and 2.6% respectively.

### Opioid Consumption

**FIGURE 1:**
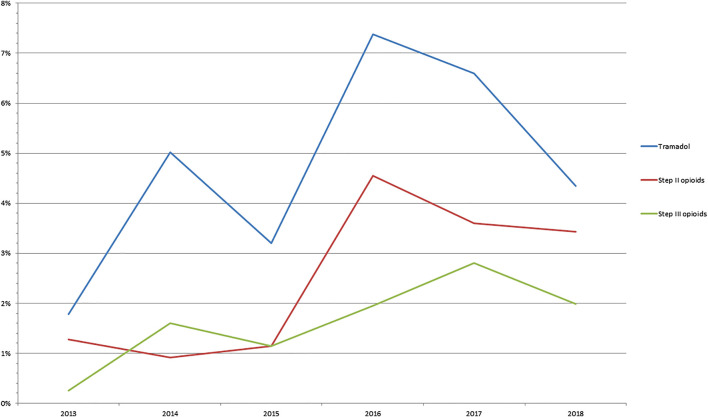
Prevalence of opioid consumption between 2013 and 2018 in patients referred for functional gastrointestinal disorders.

The global consumption of tramadol, step II and step III opioids was 4.7%, 2.7% and 1.6%, respectively in our cohort. Rheumatologic, neurologic comorbidities and migraine were more frequent in opioid patients in comparison with opioid-free patients (33.2% vs. 8.3%, 8.6% vs. 5.8% and 3.3% vs. 1.0%, *p* < 0.0001). Opioid consumption between 2013 and 2019 is presented in Figure 1 ***.*** Consumption of tramadol, step II and step III opioids significantly increased between 2013 and 2018 (*p* < 0.03, Figure 1). There were more female patients in the three opioid groups in comparison with the opioid-free group (Table 1). Patients with tramadol and step II opioids were overweight in comparison with opioid-free patients (Table 1). Other characteristics are presented in Table 1.

**TABLE 1:** Patients' characteristics according to opioid consumption.

	Tramadol (*n* = 139)	Step II opioid (*n* = 78)	Step III opioid (*n* = 47)	Opioid-free (*n* = 2689)	*p*-Value
Age (years)	55.0 [41.0; 67.0]^b^	49.0 [41.0; 62.0]	49.0 [44.0; 60.0]	50.0 [36.0; 62.0]	0.04
BMI (kg/m^2^)	25.2 [22.0; 29.3]^b^	27.7 [23.2; 32.0]^c^	24.5 [21.3; 31.2]	23.9 [21.0; 27.7]	<0.0001
Female	112 (80.6)	65 (83.3)	38 (80.8)	1941(72.2)	0.0137
**FGID** ^a^ Only IBS Only FD Both Other FGID	10.7% 41.1% 35.7% 12.5%	15.8% 31.6% 26.3% 26.3%	13.0% 34.8% 26.1% 26.1%	12.6% 39.4% 24.1% 23.8%	0.6324

**Results:** are presented in median [Q1; Q3] and *n* (percentage). BMI, body mass index; FD, functional dyspepsia; FGID, functional gastrointestinal disorder; IBS, irritable bowel syndrome. Kruskal-Wallis test and Dunn’s test were performed to compare continuous variables and chi-squared tests to compare prevalence.

a filed by 37.4% of the population

b *p* value <0.05 in comparison with opioid-free patients.

c *p* value <0.0001 in comparison with opioid-free patients.

The consumption of tramadol, step II opioids and step III opioids was 15.0%, 8.5% and 5.2% in patients with rheumatologic comorbidities, 5.6%, 2.2% and 0.6% in patients with neurologic comorbidities, and 8.9%, 13.3% and 0% in patients with migraine. Patients on opioid were also significantly more frequently on other drugs treating or affecting the GI tract in comparison with opiod-free patients (Table 2).

**TABLE 2:** Patients’ medication according to opioid consumption.

	Opioid-free *n* = 2689	Opioids *n* = 264	*p*
Antiemetics *n* = 108 (3.7%)	85 (3.2%)	23 (8.7%)	0.0001
Laxatives *n* = 287 (9.8%)	247 (9.2%)	42 (15.9%)^a^	0.0016
Loperamide *N* = 149 (5.1%)	131 (4.9%)	19 (7.2%)^b^	0.0662
NSAID *n* = 77 (2.6%)	44 (1.6%)	37 (14.0%)	<0.0001
PPI *n* = 578 (19.7%)	482 (17.9)	102 (38.6%)	<0.0001
Steroids *n* = 40 (1.4%)	31 (1.2%)	10 (3.8%)	0.0038
PAMORA *n* = 9 (0.3%)	5 (0.19%)	4 (1.5%)	<0.0001

Results are presented as *n* (percentage). NSAID, Nonsteroidal anti-inflammatory drugs; PAMORA, Peripherally-Acting Mu-Opioid Receptor Antagonists; PPI, Proton pump inhibitor.

In the tramadol group:

a Laxatives were used in 18.7%.

b Loperamide in 5.8%.

### Gastrointestinal Symptoms According to Opioid Consumption

**FIGURE 2:**
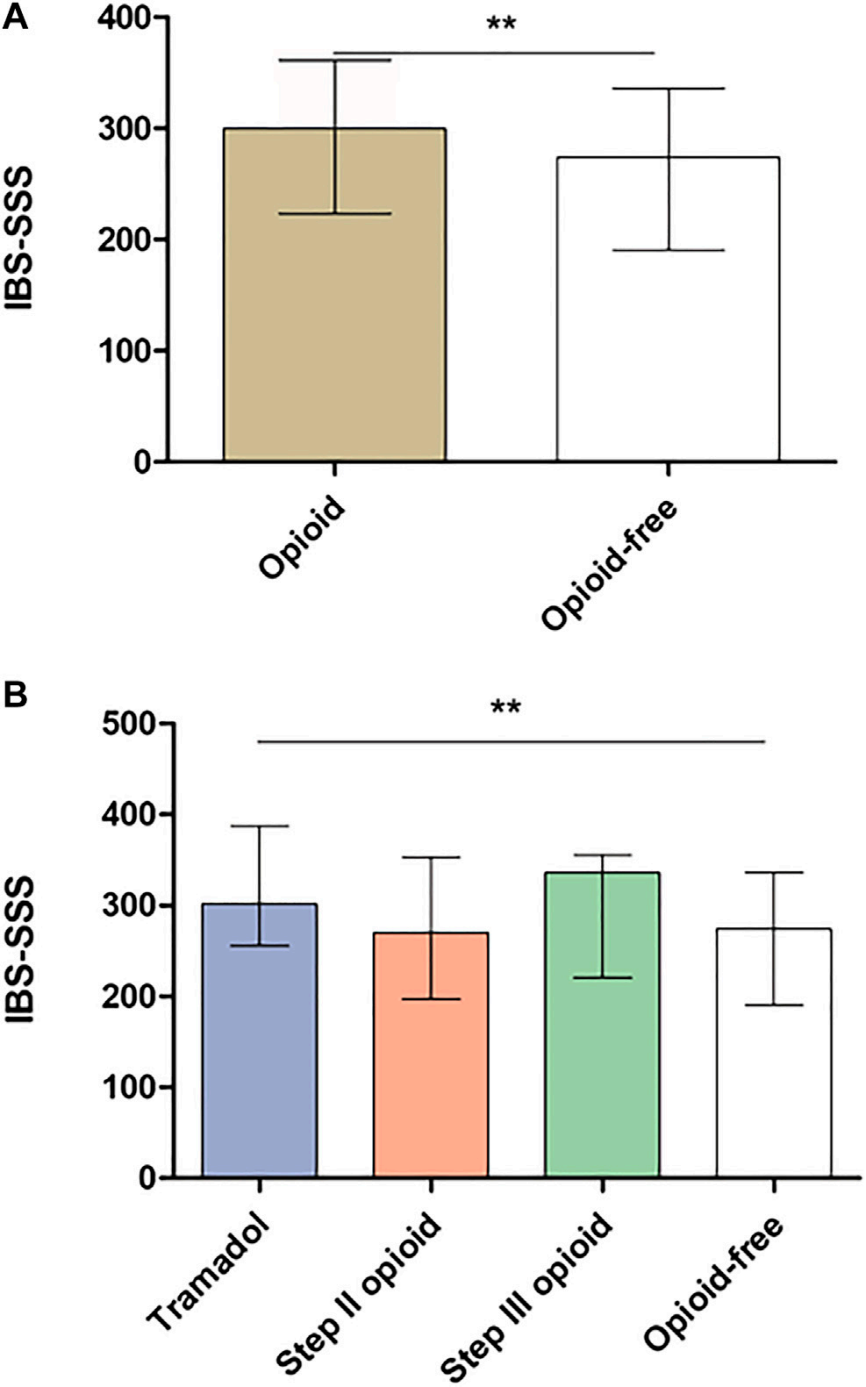
Irritable bowel syndrome severity scoring system according to opioid consumption. **(A)** IBS severity according to global opioid consumption **(B)** IBS severity according to opioid subtype. IBS-SSS: Irritable bowel syndrome severity scoring system. Results are presented in mean ± SD. **p < 0.001 in comparison with opioid-free patients. Kruskal-Wallis test and Dunn’s test were performed to compare groups of continuous variables and Mann-Whitney test to compare opioid with opioid-free groups.

**FIGURE 3:**
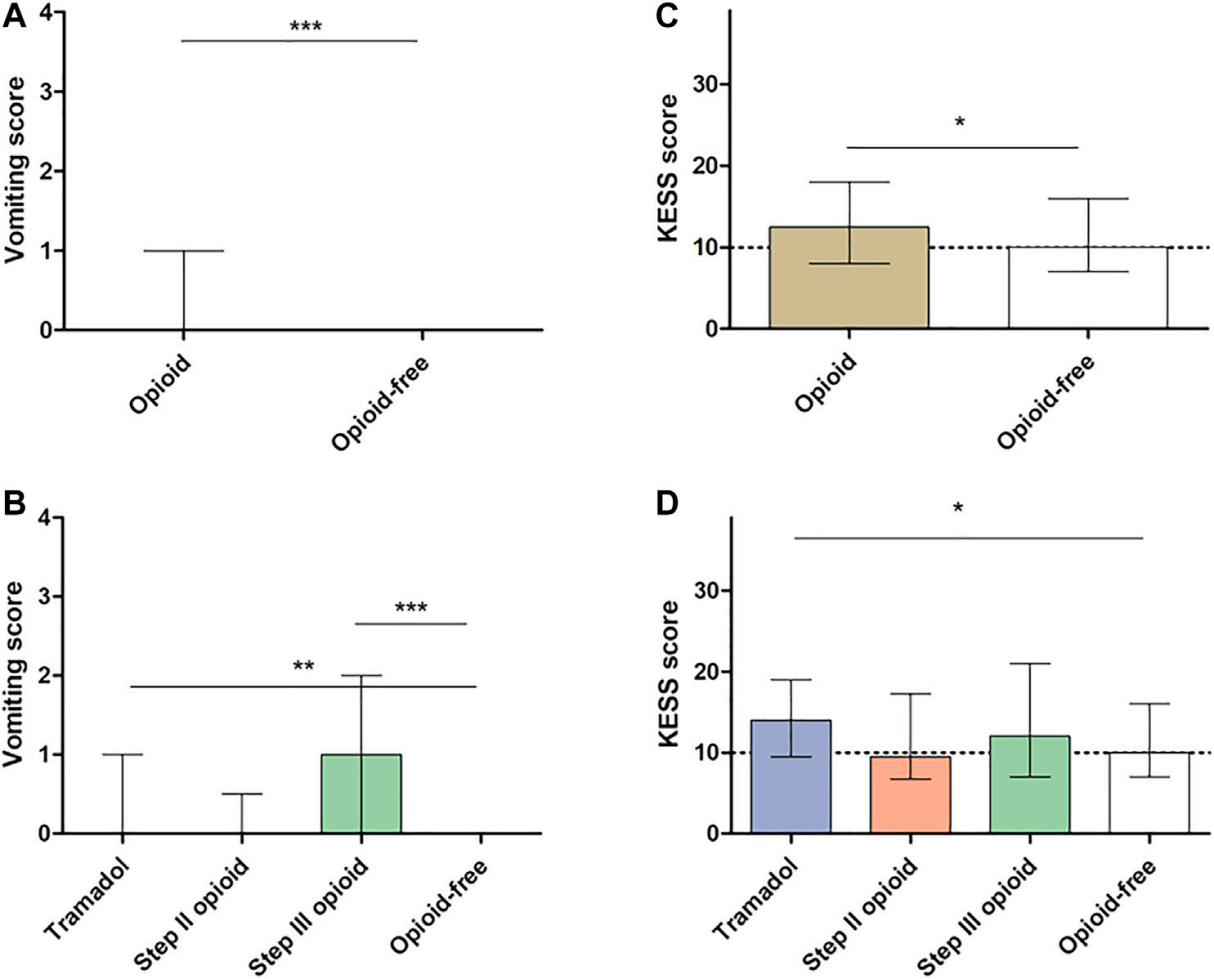
Gastrointestinal symptoms according to opioid consumption. **(A)** Vomiting score according to global opioid consumption **(B)** Vomiting score according to opioid subtype **(C,D)** Constipation score according to global opioid consumption **(C)** Constipation score according to opioid subtype **(D)**. The dotted line defines constipation (higher score) and no constipation (lower score), according to KESS definition. KESS: Knowles-Eccersley-Scott-Symptom questionnaires. Vomiting score ranges from 0 (absence of symptoms) to 4 (very severe symptoms). *p < 0.05, **p < 0.001, ***p < 0.0001 in comparison with opioid-free patients*. Kruskal-Wallis test and Dunn’s test were performed to compare groups of continuous variables and Mann-Whitney test to compare opioid with opioid-free groups.*

**FIGURE 4:**
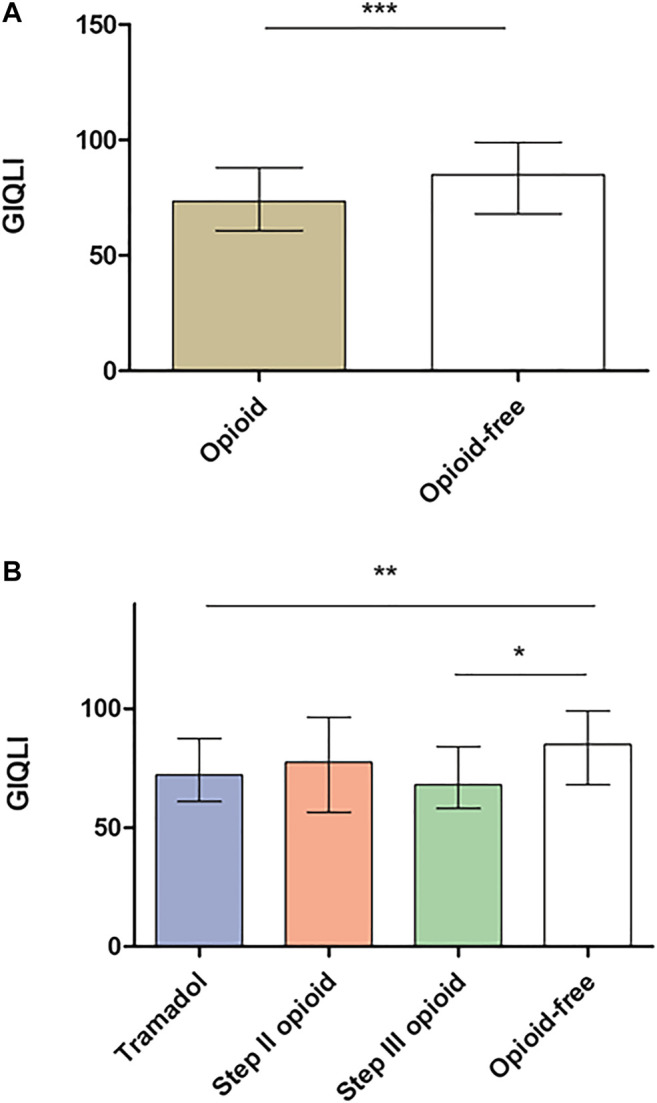
GIQLI according to opioid consumption. **(A)** Quality of life according to global opioid consumption **(B)** Quality of life according to opioid subtype. GIQLI: Gastrointestinal Quality of Life Index. Results are presented as mean ± standard deviation (SD). *p < 0.05, **p < 0.01, ***p < 0.0001 in comparison with opioid-free patients. *Kruskal-Wallis test and Dunn’s test were performed to compare groups of continuous variables and Mann-Whitney test to compare opioid with opioid-free groups.*

**FIGURE 5:**
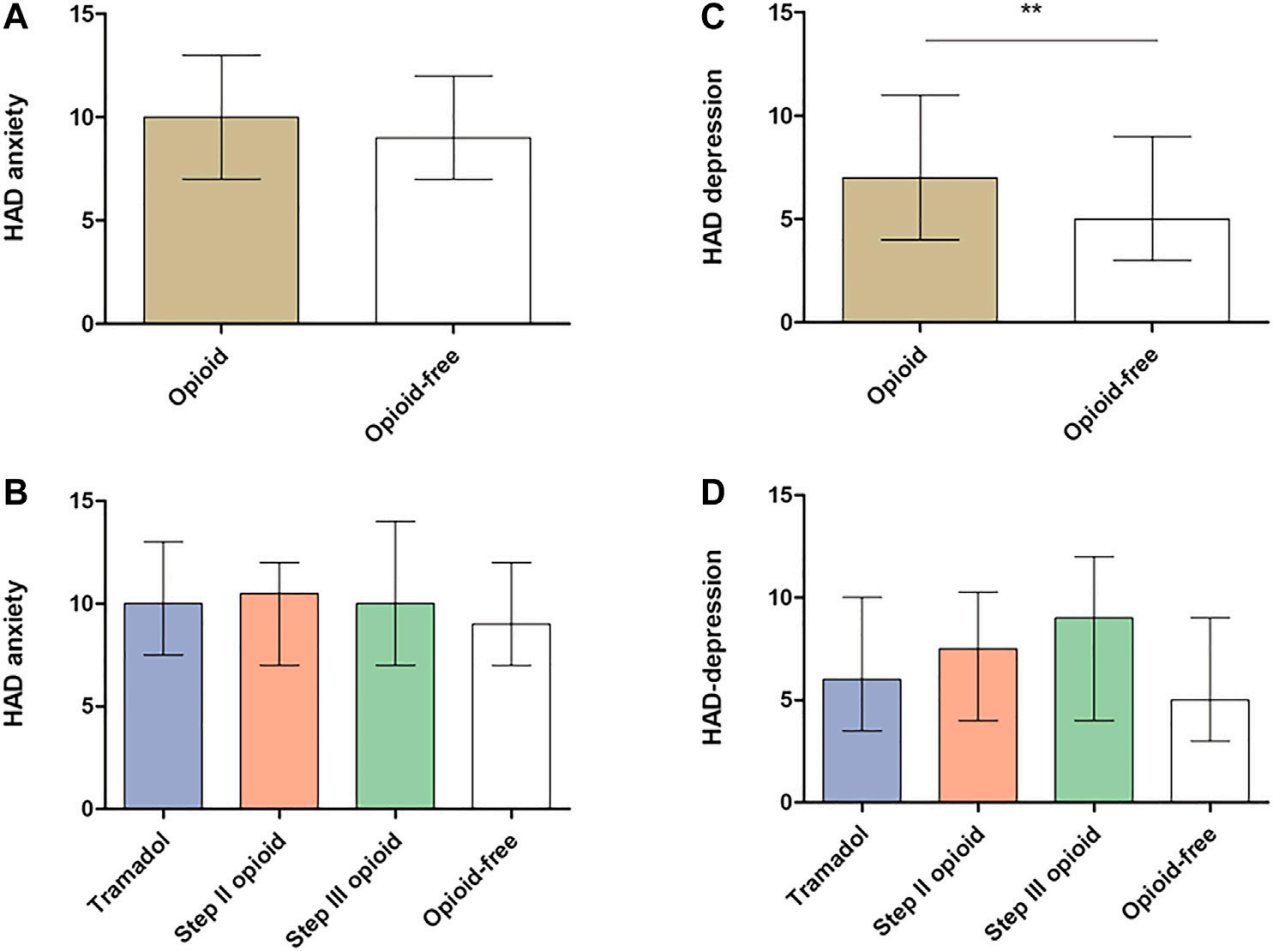
Hospital anxiety and depression scale according to opioid consumption **(A,C)** Anxiety and depression according to global opioid consumption **(B,D)** Anxiety and depression according to opioid subtype. HAD, Hospital Anxiety and Depression scale. Results are presented as mean ± standard deviation (SD). **p < 0.001. *Kruskal-Wallis test and Dunn’s test were performed to compare groups of continuous variables and Mann-Whitney test to compare opioid with opioid-free groups.*

Opioid consumption was associated with more severe GI symptoms in comparison with opioid-free patients (Figure 2A) but also with more constipation and vomiting than opioid-free patients (Figure 3A and Figure 3C). Opioid consumption was associated with a poorer quality of life (Figure 4A) and a higher depression score (Figure 5A) while anxiety was similar between opioid and opioid-free patients (Figure 5C).

Chronic consumption of tramadol was associated with more severe GI symptoms (IBS-SSS: 313.7 ± 98.2 vs. 262.5 ±104.9, *p* < 0.001, Figure 2B), with more severe constipation and vomiting (Figures 3B,D) and with a poorer quality of life (Figure 4B), in comparison with opioid-free patients. Step III opioids were associated with a higher vomiting score (Figure 3B) and a poorer quality of life (Figure 4B). Opioid subgroups were not associated with depression or anxiety (Figures 5B,D).

## Discussion

Our study is the first to assess the effect of opioid consumption in a large cohort of patients with FGID. As reported in Europe in a global population (Bosetti et al., 2019), we have shown an increase in opioid consumption over the years in FGID patients. Opioid consumption was associated with more severe GI symptoms (vomiting, constipation and GI severity), higher depression score and a poorer quality of life.

Our cohort is similar to our previously described cohort of FGID patients with middle-age female predominance (Melchior et al., 2018). Our population is typical of FGID followed in a tertiary center and well-characterized with validated questionnaires. An overlap between IBS and FD was less frequent than reported in the literature. Indeed, previous studies showed an overlap in 23%–64% of cases, according to clinical routine and tertiary center and using Rome III criteria (von Wulffen et al., 2019). The prevalence of FGID is lower using Rome IV criteria than Rome III criteria (Bai et al., 2017; Saps et al., 2018). The use of Rome IV criteria since 2016 in our cohort, may explain a lower association between IBS and FD as reported in another tertiary center (von Wulffen et al., 2019).

In our cohort, the rate of consumption of tramadol, step II opioids and step III opioids rose to 4.3%, 3.4% and 1.9% respectively and was associated with female gender. The rate of consumption of opioids was higher than in the global population and increased over the years as in other French studies (Chenaf et al., 2018; Hider-Mlynarz et al., 2018; Bosetti et al., 2019). Global consumption of opioids increased over 16% and step III opioids from 0.5% to 1.1 between 2004 and 2017 using a nationally representative sample of the French Claims database (Chenaf et al., 2018). In contrast, there was a global decrease in the consumption of step II opioids, explained by dextropropoxyphene withdrawal in 2011 in France, while consumption of mild opioids including tramadol increased (Hider-Mlynarz et al., 2018). However, opioid consumption fell after a regulatory measure in July 2017 (i.e., a medical prescription is now needed for codeine). As in our cohort, female patients were more frequently opioid users (Chenaf et al., 2018). France is in the top three countries for analgesic consumption in Europe, with paracetamol leading (Hider-Mlynarz et al., 2018). Increased opioid use is associated with increased morbidity and mortality (Chenaf et al., 2018).

Opioid consumption is associated with OIC and vomiting and altered quality of life. Indeed, in our cohort we reported more severe constipation in particular in patients treated with tramadol in comparison with opioid-free patients and higher vomiting score in both tramadol and step III opioid patients. This result cannot be explained by associated drugs, while patients with tramadol consumed more laxatives than the other group. Constipation and vomiting are both adverse events known to be induced by opioids (Els et al., 2017) and both were specifically associated with tramadol (Montastruc et al., 2018). Despite opioid efficacy, the occurrence of side effects could lead to opioid withdrawal in one third of patients (Furlan et al., 2006). OIC is the most frequent and known side effect of opioids, in particular for step III opioids (Farmer et al., 2019). OIC could be treated with laxatives which are often insufficient or PAMORA (Farmer et al., 2019). OIC prevalence was higher in patients treated with tramadol in our cohort but was not increased with step II and step III opioids. One explanation could be the weaker efficacy of PAMORA on tramadol and step II opioids (Halawi et al., 2018). The use of PAMORA to block opioid receptors is probably safe in FGID, the best option is probably to stop opioid use for these disorders but in the meantime their use in FGID warrants further studies (Corsetti et al., 2019). Another explanation could be that constipation induced by step III opioids is more recognized by clinicians and therefore probably associated with better clinical management than constipation associated with step II opioids.

Nausea and vomiting are well-known side effects of tramadol and increase with dosing (DeLemos et al., 2017). Nausea and vomiting symptoms were mostly assessed in postoperative or in cancer patients, where the causes can be various (Tsukuura et al., 2017; Shin et al., 2019). Consumption of tramadol and step III opioids was associated with a poorer quality of life in our cohort, probably linked with opioid-induced GI side effects. Mechanisms of opioid-induced nausea and vomiting are multifactorial, with central effects on the area postrema and gastric emptying modulation being key factors. Specific management of these side effects may reduce opioid withdrawal, optimize opioid efficacy and improve patients' quality of life. PAMORA efficacy has only been suggested in opioid-associated nausea and vomiting in animal models and in retrospective studies (Kanemasa et al., 2020; Sato et al., 2020). Further randomized controlled studies are therefore warranted on this particular condition.

Opioids are known to be ineffective to relieve pain symptoms in FGID (Tennant, 2015) and are therefore not a recommended therapeutic option to treat these patients. In our study, rheumatologic, neurologic comorbidities and migraine were strongly associated with opioid use, confirming that opioids in FGID patients are primarily prescribed for associated conditions. Despite their side effects, opioids are more commonly prescribed to patients with FGID than patients with identified organic gastrointestinal diseases (Sayuk et al., 2018). In addition, opioid misuse was associated with FGID in a cohort of inflammatory bowel disorders (Crocker et al., 2014; Sayuk et al., 2018). Opioids were more frequently prescribed in presence of personality disorders, psychiatric diagnoses, history of drug abuse, homelessness and/or IBS (Long et al., 2011; Sayuk et al., 2018). This association could explain the increase in depression score in the opioid group. But we have to consider that HADS is a questionnaire to screen anxiety and depression, and the diagnosis should be confirmed by a specialist, which was not the case in our study. Since 2013 in the US, gastroenterologists have prescribed fewer opioids in response to the opioid epidemic (Chen et al., 2020). In our cohort, the increased trend of opioid consumption is not likely to originate from gastroenterologists as opioid prescription was strongly associated with other GI conditions, including rheumatologic or neurologic comorbidities. Nevertheless, gastroenterologist-related initiation of opioid treatment remains to be assessed in Europe.

Of course, our study has several limitations mostly due to the retrospective analysis. The cumulative dose and duration of the opioids were not available and therefore not analyzed.

We searched for associated drugs that may have had an effect on the GI tract, but it is difficult to know whether these drugs affected our results. Indeed, the dosage and duration of all these drugs were not available for all the patients and therefore not analyzable. We have no information regarding the condition for which the drug was prescribed. Another limitation is the possibility that some patients used over the counter drugs. In the United States, half of patients with constipation used over the counter medication and only two patients out of five sought the advice of a healthcarer (Oh et al., 2020). In France many laxatives can be used without prescription, to avoid this problem we looked for all reported treatment in the medical chart but patients may have omitted to report some of them.

## Conclusion

In FGID, opioid consumption has more than tripled over a 5 year period and is higher than in the global population. Opioid consumption is associated with more severe GI symptoms (vomiting, constipation and GI severity), higher depression score and more altered quality of life. Opioid prescription should take into account worsening of GI symptoms and quality of life.

## Data Availability Statement

The original contributions presented in the study are included in the article/Supplementary Material, further inquiries can be directed to the corresponding author.

## Ethics Statement

The studies involving human participants were reviewed and approved by Rouen local human research committee (E2020-51). The patients/participants provided their written informed consent to participate in this study.

## Author Contributions

GG is the guarantor of the article. CM, FW, CD, GG, and A-ML performed the research, CM, A-ML, and GG collected and analyzed the data, AL and GG designed the research study and CM and GG wrote the paper, and CM, FW, CD, GG, A-ML, and AL contributed to the design of the study. All authors approved the final version of the article.

## Conflict of Interest

CM and GG were scientific experts for Kyowa Kirin in 2018.

The remaining authors declare that the research was conducted in the absence of any commercial or financial relationships that could be construed as a potential conflict of interest.
