# Corrigendum: Impact of Opioid Consumption in Patients With Functional Gastrointestinal Disorders Research Topic: Pharmacological Treatments Affecting Gastro-Intestinal Motility in Man

**DOI:** 10.3389/fphar.2021.652927

**Published:** 2021-04-27

**Authors:** Chloé Melchior, Charlotte Desprez, Fabien Wuestenberghs, Anne-Marie Leroi, Antoine Lemaire, Guillaume Gourcerol

**Affiliations:** ^1^ INSERM UMR 1073, Institute for Research and Innovation in Biomedicine, Normandy University, Rouen, France; ^2^ Gastroenterology Department, Rouen University Hospital, Rouen, France; ^3^ INSERM CIC-CRB 1404, Rouen University Hospital, Rouen, France; ^4^ Physiology Department Rouen University Hospital, Rouen, France; ^5^ Gastroenterology Department, CHU UCL Namur, Godinne University Hospital, UCLouvain, Yvoir, Belgium; ^6^ Pain and Palliative Care Department, Valenciennes Hospital, Valenciennes, France

**Keywords:** opioid, functional gastrointestinal disorder, tramadol, constipation, vomiting, quality of life

An author name was incorrectly spelled as Goucerol. The correct spelling is Gourcerol. The authors apologize for this error and state that this does not change the scientific conclusions of the article in any way. The original article has been updated.

